# Adaptive multiarm multistage clinical trials

**DOI:** 10.1002/sim.8464

**Published:** 2020-02-11

**Authors:** Pranab Ghosh, Lingyun Liu, Cyrus Mehta

**Affiliations:** ^1^ Pfizer Corporation Cambridge Massachusetts; ^2^ Cytel Inc Cambridge Massachusetts; ^3^ Harvard T.H. Chan School of Public Health Boston Massachusetts

**Keywords:** MAMS, early stopping, *P*‐value combination, Dunnett, adaptive Dunnett, two‐stage design, multistage design, sample size reestimation, treatment selection, FWER, pairwise comparison, closed testing, cumulative MAMS, adaptive MAMS, seamless phase 2‐3

## Abstract

Two methods for designing adaptive multiarm multistage (MAMS) clinical trials, originating from conceptually different group sequential frameworks are presented, and their operating characteristics are compared. In both methods pairwise comparisons are made, stage‐by‐stage, between each treatment arm and a common control arm with the goal of identifying active treatments and dropping inactive ones. At any stage one may alter the future course of the trial through adaptive changes to the prespecified decision rules for treatment selection and sample size reestimation, and notwithstanding such changes, both methods guarantee strong control of the family‐wise error rate. The stage‐wise MAMS approach was historically the first to be developed and remains the standard method for designing inferentially seamless phase 2‐3 clinical trials. In this approach, at each stage, the data from each treatment comparison are summarized by a single multiplicity adjusted *P*‐value. These stage‐wise *P*‐values are combined by a prespecified combination function and the resultant test statistic is monitored with respect to the classical two‐arm group sequential efficacy boundaries. The cumulative MAMS approach is a more recent development in which a separate test statistic is constructed for each treatment comparison from the cumulative data at each stage. These statistics are then monitored with respect to multiplicity adjusted group sequential efficacy boundaries. We compared the powers of the two methods for designs with two and three active treatment arms, under commonly utilized decision rules for treatment selection, sample size reestimation and early stopping. In our investigations, which were carried out over a reasonably exhaustive exploration of the parameter space, the cumulative MAMS designs were more powerful than the stage‐wise MAMS designs, except for the homogeneous case of equal treatment effects, where a small power advantage was discernable for the stage‐wise MAMS designs.

## 1 INTRODUCTION

Adaptive multiarm multistage (MAMS) clinical trials compare multiple treatment arms in pairwise fashion to a common control arm over two or more stages. These trials are characterized by interim looks at the accumulating data in order to either stop the trial early for overwhelming efficacy, stop the trial early for futilty, or to make mid‐course adaptive changes such as dropping ineffective treatment arms, changing the sample size, the error spending function, and the number of future looks. Two approaches, originating from different conceptual frameworks, have evolved for constructing adaptive MAMS designs in a statistically valid manner. We refer to them, respectively, as stage‐wise MAMS and cumulative MAMS, because of the manner in which the test statistic is constructed by each method. Although both methods may be viewed as multivariate extensions of the classical two‐arm group sequential design they differ in how they control the multiplicity inherent in an adaptive MAMS design.

The stage‐wise MAMS approach combines independent multiplicity adjusted *P*‐values from the different stages of the trial in accordance with a prespecified combination function and utilizes closed testing^1^ to ensure strong control of the family‐wise error rate (FWER). It provides full flexibility, at the end of each stage, to make data‐dependent adaptive changes, such as selecting a subset of the initial treatments or reestimating the sample size, for the remainder of the trial. Critical values for early efficacy stopping are obtained by applying the methods developed for classical two‐arm group sequential designs.^2^ Bauer and Köhne^3^ introduced this idea for two‐stage designs with multiple arms and Bauer and Kieser^4^ elaborated it further to include treatment selection at the end of stage 1. Posch et al^5^ introduced a larger family of multiplicity adjusted *P*‐values for the two stages, proposed the inverse normal combination function for combining them, and discussed parameter estimation at the end of the trial. One can directly extend this approach to *J*>2 stages, as was performed by Lehmacher and Wassmer^6^ for the special case of two‐arm trials and by Magirr, Stallard, and Jaki^7^ (Section 3.1) for multiarm trials.

The cumulative MAMS approach extends the usual two‐arm group‐sequential efficacy boundaries^2^ to the multiarm setting. A separate cumulative test statistic having an independent increments structure is obtained for the pairwise comparison of each treatment arm to a common control arm, and is monitored stage by stage. Efficacy can be claimed for any treatment arm whose statistic crosses an efficacy boundary. These efficacy boundaries are derived from the distribution of the maximum of the test statistics under the global null hypothesis that all treatment arms are ineffective. They provide strong control of the FWER. Magirr, Jaki and Whitehead^8^ generated these boundaries for the maximum of the Wald statistics. Ghosh et al^9^ reduced the computational complexity of this approach by using the maximum score statistic, in place of the maximum Wald statistic. In both these approaches, a futility boundary could be included for dropping nonperforming treatment arms at one or more stages. However, neither Reference 8 nor Reference 9 can allow for data‐dependent adaptive changes such as treatment selection or sample size reestimation. To obtain this flexibility it is necessary to incorporate both closed testing^1^ and conditional error rate methodology,^10^, ^11^ into the testing framework as was done by Koenig et al^12^ for two‐stage designs with no early stopping and by Magirr, Stallard and Jaki^7^ (Section 3.2) more generally.

This paper has two objectives. First, we show how to extend the cumulative MAMS approach of Ghosh et al^9^ to permit adaptive dose selection and sample size reestimation by use of closed testing and preservation of conditional error rates. Our approach is similar to that of References 12 and 7, but presented within the group sequential framework of Reference 2. For completeness we also present the stage‐wise MAMS approach within the group sequential framework of Reference 2, pointing out how it differs with respect to test statistics and group sequential boundaries from the cumulative MAMS approach. Second, we compare the operating characteristics of the cumulative MAMS and stage‐wise MAMS approaches, both analytically and empirically, in several settings. It is seen that the cumulative MAMS designs outperform the stage‐wise MAMS designs with respect to power in every setting but one, where there is a small, practically negligible, power advantage for the stage‐wise MAMS design. While two‐stage designs are by far the most common application of adaptive designs we have also included results for three‐stage designs. These results were previously unavailable due to the heavy computational burden they impose. The computational methods developed by Ghosh et al^9^ were essential for simulating the three‐stage cumulative MAMS designs in a realistic amount of time and thereby evaluating their operating characteristics.

In Section 2 we introduce the cumulative MAMS approach, explain how the group sequential boundaries are obtained from the distribution of the maximum score statistic, and show how to incorporate adaptive treatment selection and sample size reestimation into the design. In Section 3 we review the stage‐wise MAMS approach for making adaptive changes to an ongoing study. For ease of exposition we confine our discussion in these sections to two‐stage designs, as this suffices to explain the main principles of cumulative MAMS and stage‐wise MAMS adaptation. The more general case of *J*>2 stages is discussed in Appendix. In Section 4 we compare the power of the cumulative and stage‐wise MAMS approaches—analytically for two active doses vs placebo, and by simulation for three three active doses vs placebo. A more general simulation‐based comparison that incorporates, treatment selection, early stopping, and sample size reestimation is presented in Section 5 for a recently completed cardiovascular trial.^13^ We summarize our findings in Section 6 along with some recommendations for the choosing between the two approaches.

## 2 THE CUMULATIVE MAMS APPROACH

Consider a trial in which *D* treatment arms, indexed by *i*=1,2,…*D*, are each compared to a common control arm indexed by *i*=0. Patients are randomized to either treatment arm *i* or to the control arm in accordance with a prespecified allocation ratio λ_*i*_. We assume that a patient's response on arm *i* is normal with mean μ_*i*_ and variance $\sigma_{i}^{2}$. Let δ_*i*_=μ_*i*_−μ_0_,*i*=1,2,…*D*, represent the mean effect of treatment arm *i* relative to the control arm. Let $H_{0}^{i}:\delta_{i}=0$ denote the null hypothesis for treatment arm *i* and let $H_{0}=\cap_{i=1}^{D}H_{0}^{i}$ denote the global null hypothesis. In this section we will develop the cumulative MAMS approach for a two‐stage adaptive design to test *H*_0_ against the one‐sided alternative that δ_*i*_>0 for at least one *i*. The generalization to *J*>2 stages is presented in Appendix A1.

Let *j*=1,2 denote the first and second stages, respectively, and let *n*_*ij*_ be the sample size of arm *i* at stage *j*. Define the score statistic $W_{ij}={\hat{\delta }}_{ij}{ℐ}_{ij}$, where ${\hat{\delta }}_{ij}$ is the maximum likelihood estimate of δ_*i*_ and ${ℐ}_{ij}=n_{0j}{\left(\sigma_{0}^{2}+\lambda_{i}^{-1}\sigma_{i}^{2}\right)}^{-1}$ is its Fisher information from data up to and including stage *j*. Then ${\underline{W}}_{j}=(W_{1j},W_{2j},...W_{Dj})$ is a multivariate Brownian process with $E(W_{ij})=\delta_{i}{ℐ}_{ij}$, $\mathrm{var}(W_{ij})={ℐ}_{ij}$, $\text{cov}(W_{i1},W_{i2})={ℐ}_{i1}$, and $\text{cov}(W_{i_{1}j},W_{i_{2}j})=\Lambda_{i_{1}}\Lambda_{i_{2}}\sigma_{0}^{2}n_{0j}$ where $\Lambda_{i}={\left(\sigma_{0}^{2}+\lambda_{i}^{-1}\sigma_{i}^{2}\right)}^{-1}$. These results hold exactly if the patient level data are normally distributed and asymptotically otherwise.^14^

Let $\underline{\delta }=(\delta_{1},\delta_{2},\ldots \delta_{D})$ and $\max\{{\underline{W}}_{j}\}=\max(W_{ij},i=1,2,\ldots D)$. For future reference let $W_{i(2)}={\hat{\delta }}_{i(2)}{ℐ}_{i(2)}$ be the score statistic for the incremental data accumulated between stage 1 and stage 2, where ${ℐ}_{i(2)}=n_{0(2)}{\left(\sigma_{0}^{2}+\lambda_{i}\sigma_{i}^{2}\right)}^{-1}$ and *n*_0(2)_=*n*_02_−*n*_01_. Then ${\underline{W}}_{\left(2\right)}=(W_{1(2)},W_{2(2)},\ldots W_{D(2)})$ is independent of ${\underline{W}}_{1}$ and has a multivariate normal distribution with $E(W_{i(2)})=\delta_{i}{ℐ}_{i(2)}$, $\mathrm{var}(W_{i(2)})={ℐ}_{i(2)}$, and $\text{cov}(W_{i_{1}(2)},W_{i_{2}(2)})=\Lambda_{i_{1}}\Lambda_{i_{2}}\sigma_{0}^{2}n_{0(2)}$. In practice, when evaluating these distributions, we will replace the unknown Fisher information quantities ${ℐ}_{i1},{ℐ}_{i2}$ and ${ℐ}_{i(2)}$ by corresponding estimates, ${\hat{ℐ}}_{i1},{\hat{ℐ}}_{i2},\text{and}{\hat{ℐ}}_{i(2)},$ from the data. (See, for example, equation (9)). The simulation results in Table 1 of Section 5 demonstrate that this second‐order approximation preserves type‐1 error even for relatively small sample sizes. Using computational methods discussed in Ghosh et al^9^ for multivariate Brownian processes we can obtain level‐α group sequential boundaries (*b*_1_,*b*_2_) such that

$$P_{\underline{0}}(\max\{{\underline{W}}_{1}\}\geq b_{1})=\alpha_{1}\;\text{and}\;P_{\underline{0}}(\max\{{\underline{W}}_{1}\}<b_{1}\cap \max\{{\underline{W}}_{2}\}\geq b_{2})=\alpha -\alpha_{1},$$

where $P_{\underline{h}}(.)$ denotes probability under $\underline{\delta }=\underline{h}$ and α_1_ is the portion of the prespecified allowable type‐1 error that is spent at stage 1.

**Table 1:** Power comparisons of single stage, stage‐wise multiarm multistage (MAMS) and cumulative MAMS designs

(A) Two‐stage SOCRATES design (10 000 simulated trials)					
	Power (standard error)				
	Single	Adaptive Stage‐Wise MAMS			Adaptive
	Stage				Cumulative
$\underline{\delta }$ (with σ=0.52)	Dunnett	Bonferroni	Simes	Dunnett	MAMS
(0.187, 0.187, 0.187)	0.804 (.004)	0.728 (.004)	0.785 (.004)	0.786 (.004)	0.805 (.004)
(0, 0.187, 0.187)	0.731 (.004)	0.667 (.005)	0.713 (.004)	0.734 (.004)	0.768 (.004)
(0, 0, 0.187)	0.591 (.005)	0.521 (.005)	0.527 (.005)	0.597 (.005)	0.657 (0.005)
(0, 0, 0)	0.025 (.002)	0.018 (.001)	0.020 (.001)	0.021 (.001)	0.023 (.001)
Drop any treatment *i* at stage 1 if corresponding ${\hat{\delta }}_{i1}<0$					
**(B) Three‐stage SOCRATES design (10 000 simulated trials)**					
	**Power (SE)**				
	**Single**	**Adaptive Stage‐Wise MAMS**			**Adaptive**
	**Stage**				**Cumulative**
$\underline{\delta }$ **(with σ=0.52**)	**Dunnett**	**Bonferroni**	**Simes**	**Dunnett**	**MAMS**
(0.187, 0.187, 0.187)	0.804 (.004)	0.678 (.005)	0.778 (.004)	0.787 (.004)	0.806 (.004)
(0, 0.187, 0.187)	0.731 (.004)	0.610 (.005)	0.691 (.005)	0.725 (.004)	0.773 (.004)
(0, 0, 0.187)	0.591 (.005)	0.445 (.005)	0.494 (.005)	0.592 (.005)	0.647 (.005)
(0, 0, 0)	0.025 (.002)	0.017 (0.001)	0.018 (.001)	0.022 (.001)	0.023 (.001)
Drop any treatment *i* at stage 1 if corresponding ${\hat{\delta }}_{i1}<0$					

We shall, throughout, denote observed values of random variables by lowercase letters. Thus ${\underline{w}}_{1}$ denotes the observed value of ${\underline{W}}_{1}$. We may reject any hypothesis $H_{0}^{i}$ for which the corresponding *w*_*i*1_≥*b*_1_. The trial is then terminated for efficacy. If, however, $\max\{{\underline{w}}_{1}\}<b_{1}$ the trial continues to stage 2 where again any hypothesis $H_{0}^{i}$ is rejected for which the corresponding *w*_*i*2_≥*b*_2_. Due to the use of the max statistic this hypothesis testing procedure maintains strong control of the FWER.^8^

It is important to recognize that the efficacy boundaries for a multiarm group sequential design must be stricter than the corresponding efficacy boundaries for a two‐arm group sequential design, since the former have to adjust for the multiplicity due to testing more than one hypothesis at each look. For example, if *D*=4 the multiarm group sequential boundaries for treatment *i*, derived from the Lan and DeMets^15^ error spending function are $b_{1}=3.3453\sqrt{{ℐ}_{i1}}$ and $b_{2}=2.4510\sqrt{{ℐ}_{i2}}$ for a one‐sided test at α=0.025 and an interim look at 50% of the total information. In contrast the two‐arm group sequential efficacy boundaries in this setting are $b_{1}=2.9626\sqrt{{ℐ}_{i1}}$ and $b_{2}=1.9686\sqrt{{ℐ}_{i2}}$.

We consider two possible adaptations at the end of stage 1. (a) Permit one or more treatment arms to be dropped. (b) Alter the sample size of each treatment arm *i* that will be proceeding to stage 2, while maintaining its allocation ratio λ_*i*_. Strong control of FWER can be maintained without any adjustment to the group sequential design if (a) is the only adaptation. We can, optionally, improve the efficiency of the design by recomputing the stage 2 boundary in conjunction with closed testing. If, on the other hand, the adaptation includes (b) then it is essential to recompute the stage 2 boundary in conjunction with closed testing in order to maintain strong control of FWER. We next discuss how this is accomplished.

Let 𝒟={1,2,…D} and S⊆𝒟 denote the indices of the treatments selected for stage 2. At stage 2 we are interested in testing $H_{0}^{i}$ for all *i*∈*S* while maintaining strong control of the FWER at level α. To achieve this control, each $H_{0}^{i}$ must be tested by a closed level‐α test. That is, $H_{0}^{i}$ may only be rejected if, for all I⊆𝒟 such that *i*∈*I*, $H_{0}^{I}=\cap_{g\in I}H_{0}^{g}$ is rejected with a valid local level‐α test.^1^ The valid local level‐α test of $H_{0}^{I}$ is constructed in two steps.

Step 1 Compute two‐stage group sequential level‐α boundaries (*b*_*I*1_,*b*_*I*2_) for making ||*I*|| comparisons to a common control. These boundaries must satisfy
  (1) $$P_{\underline{0}}(\max\{{\underline{W}}_{I1}\;\}\geq b_{I1})=\alpha_{1}\;\text{and}\;P_{\underline{0}}(\max\{{\underline{W}}_{I1}<b_{I1}\cap \max\{{\underline{W}}_{I2}\}\geq b_{I2}\})=\alpha -\alpha_{1},$$
  where ${\underline{W}}_{Ij}=\{W_{gj};g\in I\}$, *j*=1,2. If $\max\{{\underline{W}}_{I1}\}\geq b_{I1}$, $H_{0}^{I}$ is rejected. Otherwise we proceed to Step 2.
Step 2 After examining the stage 1 data a subset S⊆𝒟 consisting of ||*S*|| treatments is selected for testing at stage 2. Suppose that the incremental stage 2 sample size of the control arm is altered from *n*_0(2)_ to $n_{0(2)}^{*}$, and suppose that the incremental stage 2 sample sizes of the ||*S*|| treatment arms are correspondingly increased so as to preserve their respective allocation ratios relative to the control arm. Let *I*_*S*_=*I*∩*S*. In order to preserve the type‐1 error of the trial we must replace the stage 2 boundary *b*_*I*2_ with $b_{I2}^{*}$ such that
  (2) $$P_{0}(\max\{{\underline{W}}_{I_{S}2}^{*}\}\geq b_{I2}^{*}|{\underline{w}}_{I1})=P_{0}(\max\{{\underline{W}}_{I2}\}\geq b_{I2}|{\underline{w}}_{I1}),$$
  where ${\underline{W}}_{I_{S}2}^{*}=\{W_{g2}^{*}:g\in I_{S}\}$ and the *“*∗*”* indicates that the sample size of the stage 2 statistic $W_{g2}^{*}$ has been altered from *n*_*g*2_ to $n_{g2}^{*}=n_{g1}+n_{0(2)}^{*}\lambda_{g}$. We reject $H_{0}^{I}$ if $\max\{{\underline{W}}_{I_{S}2}^{*}\}\geq b_{I2}^{*}$. Equation (2) is a consequence of the conditional error rate principle^11^ which states that in order to preserve the overall type‐1 error of the trial its conditional type‐1 error after adaptation should not exceed the conditional type‐1 error of the original trial, given the stage 1 data. Thereby $H_{0}^{I}$ is rejected by a valid level‐α test.

Finally, rejection of $H_{0}^{i}$ requires that $H_{0}^{I}$ be rejected in the above manner for all possible subsets I⊆𝒟 that contain i. This will ensure that the test of $H_{0}^{i}$ is closed and will thereby guarantee strong control of FWER.

## 3 THE STAGE‐WISE MAMS APPROACH

We recapitulate the two‐stage method described by Reference 5, but present it in the classical group sequential framework of Reference 2, which facilitates generalization to *J*>2 stages as given in Appendix A2. Recall from Section 2 that we can reject any elementary hypothesis $H_{0}^{i}$ only if the intersection hypothesis $H_{0}^{I}$ is rejected by a valid local level‐α test for all subsets I⊆𝒟 that contain *i*. In stage‐wise MAMS the test of $H_{0}^{I}$ utilizes multiplicity adjusted *P*‐values computed from the *incremental* data at stages 1 and 2. Any valid multiplicity adjusted *P*‐values may be utilized for this purpose. Popular candidates include the *t*‐test based *P*‐values adjusted for multiplicity by the nonparametric Bonferroni and Simes procedures for which the appropriate formulae are given in Reference 5. However, in order to make a meaningful comparison between the cumulative and stage‐wise MAMS approaches, we will utilize *P*‐values that are derived from the maximum score statistic. In that case the multiplicity adjusted *P*‐value for testing $H_{0}^{I}$ at stage *j* is the single‐stage Dunnett *P*‐value^16^

(3) $$p_{I(j)}=P_{H_{0}^{I}}\left(\max\{{\underline{W}}_{I(j)}\}\geq \max\{{\underline{w}}_{I(j)}\}\right),$$

where ${\underline{W}}_{I(1)}$ and ${\underline{W}}_{I(2)}$ are the score statistics based on the incremental data at stages 1 and 2, respectively. To evaluate Equation (3) exactly we define, for all *i*∈*I*,

$$t_{i(j)}=\frac{w_{i(j)}}{\sqrt{{\hat{ℐ}}_{i(j)}}},$$

where ${\hat{ℐ}}_{i(j)}$ is the estimated Fisher information from the incremental data of stage j. Define ${\underline{t}}_{I(j)}=\{t_{i(j)};i\in I\}$. Then the multiplicity adjusted Dunnett *P*‐value can be computed exactly as

(4) $$p_{I(j)}=P_{H_{0}^{I}}\left(\max\{{\underline{T}}_{I(j)}\}\geq \max\{{\underline{t}}_{I(j)}\}\right),$$

where ${\underline{T}}_{I(j)}$ has a multivariate‐*T* distribution with mean $\underline{0}$, $n_{0(j)}+\sum_{i\in I}n_{i(j)}-||I||-1$ degrees of freedom, and a known covariance matrix that depends on the allocation ratios of the treatment arms to the control arm.

A two‐stage level‐α test of $H_{0}^{I}$ can now be constructed as follows. Define the test statistic for stage 1 as

$$Z_{I1}=\Phi^{-1}(1-p_{I(1)})\;.$$

We will use the same type‐1 error, α_1_, for stage 1 as was used in the cumulative MAMS approach. Thus for any I⊆𝒟, $H_{0}^{I}$ is rejected by a valid level‐α_1_ test if *Z*_*I*1_≥*c*_1_, where *c*_1_= Φ^−1^(1−α_1_). The trial terminates for efficacy at stage 1 if there exists at least one i∈𝒟 such that for all I⊆𝒟 that contain *i*, *Z*_*I*1_≥*c*_1_, for then $H_{0}^{i}$ can be rejected by a level‐α_1_ closed test.

If the trial does not terminate at stage 1 let S⊆𝒟 be the set of treatment indexes selected for stage 2 and *I*_*S*_=*I*∩*S* be the set of treatments from *I* that are carried forward to stage 2. Let $\max\{{\underline{W}}_{I_{S}(2)}\}=\max(W_{q(2)};q\in I_{S})$ denote the maximum incremental score statistic in the set *I*_*S*_. Then the second‐stage *P*‐value for testing $H_{0}^{I}$ is

(5) $$p_{I(2)}=P_{\underline{0}}(\max\{{\underline{T}}_{I_{S}(2)}\}\geq \max\{{\underline{t}}_{I_{S}(2)}\}).$$

We now compute the test statistic for stage 2 as a weighted sum of inverse normal components

$$Z_{I2}=h_{1}\Phi^{-1}(1-p_{I(1)})+h_{2}\Phi^{-1}(1-p_{I(2)})\;,$$

where *h*_1_ and *h*_2_ are prespecified weights whose sum of squares is 1. The statistics *Z*_*I*1_ and *Z*_*I*2_ are *N*(0,1) under $H_{0}^{I}$ and *Z*_*I*2_−*Z*_*I*1_ is independent of *Z*_*I*1_. Thus one can readily obtain the efficacy boundary *c*_2_ such that

$$P_{H_{0}^{I}}(Z_{I1}<c_{1}\cap Z_{I2}\geq c_{2})=\alpha -\alpha_{1},$$

by the usual methods for two‐arm group sequential designs.^2^ We reject $H_{0}^{i}$ with strong control of FWER if *Z*_*I*2_≥*c*_2_ for all possible I⊆𝒟 with *i*∈*I*. The generalization to *J*>2 stages is given in Appendix A2.

Note that the efficacy boundaries (*c*_1_,*c*_2_) only protect the multiplicity induced by testing the same hypothesis over two stages. In particular, they do not adjusted for the multiplicity due to testing multiple treatment arms against a common control arm. The latter multiplicity adjustment is applied through the Dunnett *P*‐values. In contrast the cumulative MAMS approach applies the adjustments for both the sources of multiplicity directly through the efficacy boundaries. For example, if 𝒟=4 the Lan‐DeMets^15^ efficacy boundaries for the stage‐wise MAMS design are *c*_1_=2.9626 and *c*_2_=1.9868. These are the efficacy boundaries for comparing a single treatment arm to a control arm even though in fact four treatments are being compared to the same control. For the cumulative MAMS design, however, the Wald‐scale boundaries for comparing four treatments to a common control would be $b_{1}/\sqrt{{ℐ}_{i1}}=3.3453$ and $b_{2}/\sqrt{{ℐ}_{i2}}=2.4510$.

## 4 CUMULATIVE MAMS VS STAGE‐WISE MAMS

Our goal is to compare the cumulative and stage‐wise MAMS approaches with respect to global power, defined here as the probability of rejecting $H_{0}^{i}$ for any treatment *i*, *i*=1,2,…*D*. We will first make these comparisons for the special case of two active doses, no early stopping and no dose selection. In this ideal setting it is possible to make the comparisons analytically and thereby gain a deeper insight into the conditions under which one method has greater power than the other. We will then extend these comparisons to more general settings by simulation.

### 4.1 Analytical Comparison with Two Active Doses and Two Stages

Patients are randomized equally between the three arms of the study and each patient's response is normally distributed with σ^2^=1. The control arm has a mean of zero and treatment *i* has mean δ_*i*_, *i*=1,2. The null hypothesis corresponding to the treatment *i* is $H_{0}^{i}:\delta_{i}=0$. We will test the global null hypothesis $H_{0}=H_{0}^{1}\cap H_{0}^{2}$ against the one‐sided alternative that δ_*i*_>0 for at least one *i*=1,2. Under the assumption of no early stopping, no dropping of treatments and no adaptive sample size reestimation, one can derive analytical power functions for the cumulative and stage‐wise MAMS designs. Let *f*_1_(*w*_11_,*w*_21_) be the probability density function of ${\underline{W}}_{1}=(W_{11},W_{21})$, the stage 1 score statistics. Let *f*_(2)_(*w*_1(2)_,*w*_2(2)_) be the probability density function of ${\underline{W}}_{\left(2\right)}=(W_{1(2)},W_{2(2)})$, the incremental stage 2 score statistics.(For notational convenience we have suppressed the dependence of these densities on $\underline{\delta }$.) Let *b*_2_ denote the critical value for declaring statistical significance at the end of stage 2. Then we have shown in Appendix A1 that *P*(*CUMUL*) and *P*(*STAGE*), the respective cumulative and stage‐wise MAMS probabilities of rejecting *H*_0_ when the true treatment effect is $\underline{\delta }=(\delta_{1},\delta_{2})$, are given by

(6) $$P(CUMUL)=1-\overset{\infty }{\underset{-\infty }{\int }}\overset{\infty }{\underset{-\infty }{\int }}\left(\overset{b_{2}-w_{11}}{\underset{w_{1(2)}=-\infty }{\int }}\overset{b_{2}-w_{21}}{\underset{w_{2(2)}=-\infty }{\int }}f_{\left(2\right)}\left(w_{1(2)},w_{2(2)}\right)dw_{2(2)}dw_{1(2)}\right)f_{1}(w_{11},w_{21})dw_{21}dw_{11}$$

and

(7) $$P(STAGE)=1-\overset{\infty }{\underset{-\infty }{\int }}\overset{\infty }{\underset{-\infty }{\int }}\left(\overset{F_{\left(2\right)}^{-1}(g)}{\underset{w_{1(2)}=-\infty }{\int }}\overset{F_{\left(2\right)}^{-1}(g)}{\underset{w_{2(2)}=-\infty }{\int }}f_{\left(2\right)}\left(w_{1(2)},w_{2(2)}\right)dw_{2(2)}dw_{1(2)}\right)f_{1}(w_{11},w_{21})dw_{21}dw_{11}\;,$$

where $p_{1}=P_{\underline{0}}(\max\{{\underline{W}}_{1}\}\geq \max\{{\underline{w}}_{1}\})$ and $p_{\left(2\right)}=P_{\underline{0}}(\max\{{\underline{W}}_{\left(2\right)}\}\geq \max\{{\underline{w}}_{\left(2\right)}\})$ are the multiplicity‐adjusted *P*‐values for the two stages, and $g=\Phi \left(\frac{Z_{\alpha }-h_{1}Z_{p_{1}}}{h_{2}}\right)$ is a function of the maximum of (*w*_11_,*w*_21_) through *p*_1_.

It is instructive to compare the two power functions (6) and (7). They differ only in the upper limits of the inner (or stage 2) integrals. In *P*(*CUMUL*) the stage 2 score statistics (*w*_1(2)_,*w*_2(2)_) are confined to the region (−∞,*b*_2_−*w*_11_)×(−∞,*b*_2_−*w*_21_). Notice that this is the acceptance region for a test that rejects *H*_0_ if either *w*_11_+*w*_1(2)_≥*b*_2_ or *w*_21_+*w*_2(2)_≥*b*_2_. Thus *P*(*CUMUL*) is derived from a test that is based on sufficient statistics. In contrast the stage 2 score statistics (*w*_1(2)_,*w*_2(2)_) in the expression for *P*(*STAGE*) are confined to the region $\left(-\infty ,F_{\left(2\right)}^{-1}(g))\times (-\infty ,F_{2}^{-1}(g)\right)$. This is the acceptance region for a test that rejects *H*_0_ if $h_{1}z_{p_{1}}+h_{2}z_{p_{\left(2\right)}}\geq z_{\alpha }$. Clearly this test is not based on sufficient statistics.

**Figure 1:**
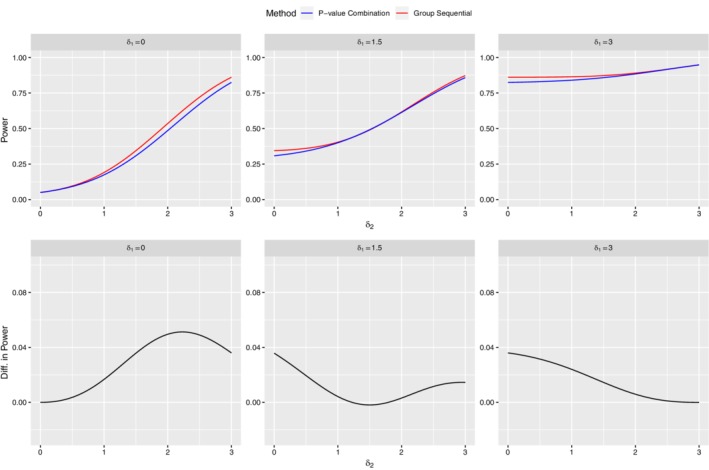
Analytical power comparisons: Stage‐wise vs cumulative multiarm multistage

**Figure 2:**
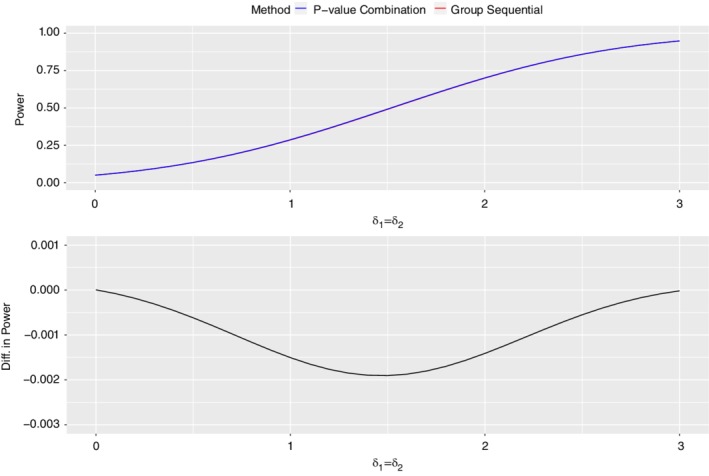
Detailed analytical power comparisons at *δ*_1_=*δ*_2_

The impact on global power of nonadherence to the sufficiency principle is shown in Figure 1, where the two‐test methods are compared for δ_1_ and δ_2_ in the range 0 to 3, and in Figure 2, where the two‐test methods are compared with equal δ values over the range δ_1_=δ_2_=0 to δ_1_=δ_2_=3. We have chosen α=0.05 for both test methods, with total statistical information ${ℐ}_{2}=1$ for evaluating *P*(*CUMUL*), and stage‐wise statistical information ${ℐ}_{1}={ℐ}_{\left(2\right)}=0.5$ for evaluating *P*(*STAGE*). With these design parameters both designs achieve 0.95 power at δ_1_=δ_2_=3 and FWER equal to 0.05 at δ_1_=δ_2_=0. The following conclusions may be drawn:

1. Except for a small region near δ_1_=δ_2_=1.5, *P*(*CUMUL*) exceeds *P*(*STAGE*) everywhere, with absolute power gains between 0% and 5%.
2. When δ_1_=δ_2_=1.5 there is a tiny power loss, *P*(*CUMUL*)−*P*(*STAGE*)=−0.2*%*, which disappears rapidly as soon as δ_2_ moves away from δ_1_.
3. The power gain for *P*(*CUMUL*) is maximum when the two δ values differ by the greatest amount; δ_1_=0,δ_2_=3 or δ_1_=3,δ_2_=0
4. The slight loss in power at δ_1_=δ_2_=1.5 shown in Figure 1 suggests that similar losses might also occur at other values of δ_1_=δ_2_. This is confirmed by an examination of Figure 2 where *P*(*CUMUL*)−*P*(*STAGE*) is plotted over the range δ_1_=δ_2_=0 to δ_1_=δ_2_=3. The power loss is zero at δ_1_=δ_2_=0, increases gradually to a maximum of −0.002 at δ_1_=δ_2_=1.5 and then declines, reaching zero once again at δ_1_=δ_2_=3.

It is worth noting that, in this setting the cumulative MAMS design has the property of consonance. When *H*_0_ is rejected by the cumulative MAMS method we can, in addition to rejecting *H*_0_, also reject either $H_{0}^{1}$ or $H_{0}^{2}$ or both of them, depending on which component(s) of ${\underline{w}}_{2}$ crossed the efficacy boundary. For the *P*‐value combination test, however, rejecting *H*_0_ does not provide any additional information about the status of $H_{0}^{1}$ or $H_{0}^{2}$ individually. We need to further reject either $H_{0}^{1}$ or $H_{0}^{2}$ or both by local level‐α tests before we an make an efficacy claim for these dose groups. These additional tests have not been factored into the analytical power calculations for the *P*‐value combination approach. Therefore we can conclude that the actual power of the *P*‐value combination approach to identify efficacious doses is even less than *P*(*STAGE*).

### 4.2 Simulation‐based comparison with three active doses and selection

The analytical expressions in Equations (6) and (7) were derived in the idealized setting of two active doses, no early stopping and no dropping of treatment arms at the end of stage 1. We now consider the more realistic setting of three active doses in which nonperforming doses are dropped at the end of stage 1.

**Figure 3:**
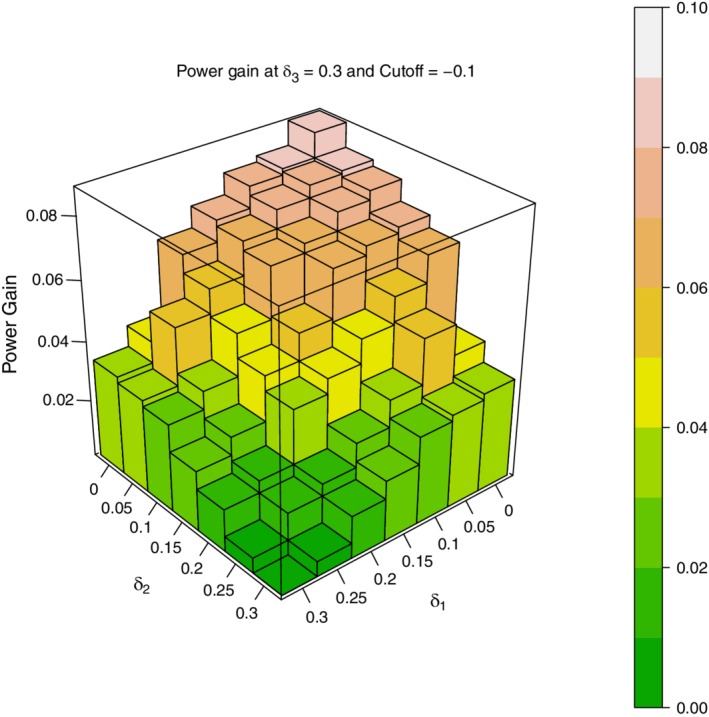
*P*(*CUMUL*)−*P*(*STAGE*): *δ*_3_=0.3; (*δ*_1_,*δ*_2_)=0,(0.5),0.3; drop dose if *δ*_*i*_<−0.1

**Figure 4:**
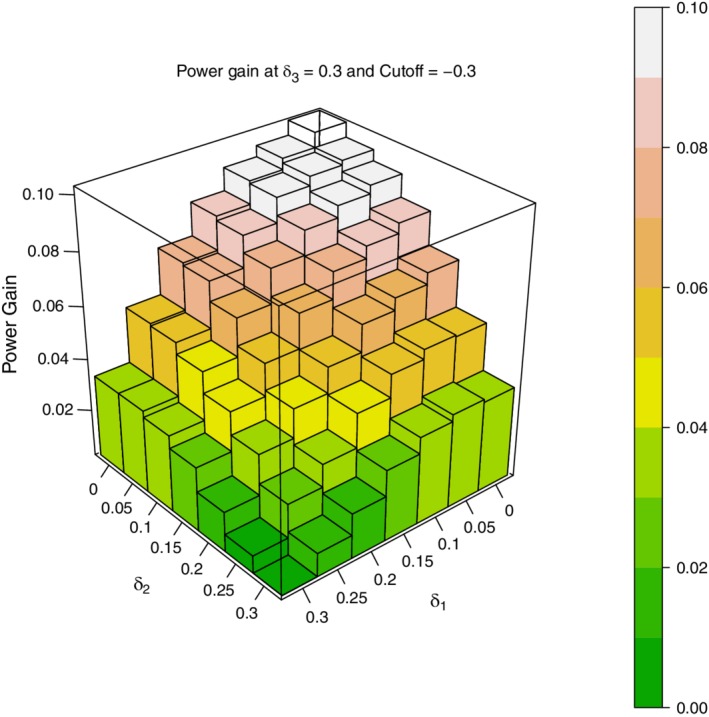
*P*(*CUMUL*)−*P*(*STAGE*): *δ*_3_=0.3; (*δ*_1_,*δ*_2_)=0,(0.5),0.3; drop dose if *δ*_*i*_<−0.3

Figure 3 is a three‐dimensional (3D) plot showing the absolute power gain, *P*(*CUMUL*)−*P*(*STAGE*), when δ_3_=0.3 , (δ_1_,δ_2_)=0,0.05,…,0.3, σ^2^=1, and treatment *i* is dropped at the end of stage 1 if ${\hat{\delta }}_{i}<-0.1$. Figure 4 is a similar 3D plot with the same σ^2^ and range of values for the δ's, but with a stricter criterion for dropping doses; here treatment *i* is dropped if ${\hat{\delta }}_{1}<-0.3$. Both plots are based on 10 000 simulated trials. By examining these plots one may draw three important conclusions about the power differential between the cumulative MAMS and stage‐wise MAMS designs.

1 *P*(*CUMUL*) exceeds *P*(*STAGE*) with absolute power gains up to 9% when the cut‐off for dropping doses is ${\hat{\delta }}_{i}<-0.1$ and up to 11% when the cut‐off for dropping doses is ${\hat{\delta }}_{i}<-0.3$
2 The gain in power of *P*(*CUMUL*) over *P*(*STAGE*) appears to depend on the degree of heterogeneity among the δ values. The greater the heterogeneity, the greater the power gain. To see this note the following:
  - The gain in power of *P*(*CUMUL*) over *P*(*STAGE*) is maximum when δ_1_=δ_2_=0 and δ_3_ = 0.3
  - The gain in power of *P*(*CUMUL*) over *P*(*STAGE*) is zero when δ_1_=δ_2_=δ_3_=0.3
  - At δ_3_=0.3 and any fixed value for δ_1_, the gain in power of *P*(*CUMUL*) over *P*(*STAGE*) increases as δ_2_ decreases from 0.3 to 0.
  - At δ_3_=0.3 and any fixed value for δ_2_, the gain in power of *P*(*CUMUL*) over *P*(*STAGE*) increases as δ_1_ decreases from 0.3 to 0
3. The gain in power of *P*(*CUMUL*) over *P*(*STAGE*) is larger in Figure 4 than in Figure 3 for every (δ_1_,δ_2_,δ_3_) combination. As the only difference between the two figures is the value of ${\hat{\delta }}_{i}$ below which doses are dropped, it would appear that the stricter the criterion for dropping doses at the end of stage 1, the greater the power differential. We will revisit this conjecture in Section 5 in the context of an actual clinical trial.

Figures 3 and 4 display results only for the portion of the parameter space where δ_3_=0.3 and (δ_1_,δ_2_)≤δ_3_. For completeness, additional simulations were also carried out in the region of the parameter space where δ_1_ and δ_2_ exceed δ_3_=0.3. Here too *P*(*CUMUL*) exceeded *P*(*STAGE*) everywhere. The power gains were, however, small (about 0.5% on average), because in this region of the parameter space, both *P*(*CUMUL*) and *P*(*STAGE*) had very large absolute powers—93% to 99%.

## 5 THE SOCRATES‐REDUCED TRIAL

SOCRATES‐REDUCED was a multicenter, randomized, placebo‐controlled trial which enrolled patients with worsening chronic heart failure after clinical stabilization.^13^ Patients were randomized to three different dose groups (2.5, 5, and 10 mg) of oral vericiguat or placebo. The primary end point of the trial was change from baseline to week 12 in log‐transformed N‐terminal pro‐B‐type natriuretic peptide (NT‐proBNP). The statistical analysis plan specified that for the analysis of the primary endpoint the patients from the three dose groups would be pooled and compared to the placebo arm. The trial was designed for 80% power to detect a difference of δ=0.187 between the pooled dose group and placebo, at one‐sided α=0.025. In order to meet these design requirements, and assuming that σ=0.52, a total of 260 patients (65/arm) were randomized to the study. This trial, however, failed to show statistical significance. The observed treatment effect for the pooled dose group relative to placebo was only 0.122 (*P*‐value = .075, one‐sided).

The data from the trial showed a dose‐response relationship with an observed difference from placebo of 0.248 for the 10‐mg dose group (*P* = .024), 0.073 for the 5‐mg dose group (*P* = .15), and 0.04 for the 2.5‐mg dose group (*P* = .19). Pooling the three dose groups for the final analysis caused a dilution of the observed treatment effect and resulted in a failed trial even though the 10‐mg dose appears to be clearly effective. We will use this example to display the operating characteristics of alternative cumulative and stage‐wise MAMS designs that might have been used for identifying effective doses in a multiarm setting.

A single‐stage four‐arm design based on Dunnett's test in which σ=0.52 and δ=0.187 for each dose vs placebo requires 388 patients (97/arm) for 80% power at one‐sided α=0.025. Here power is defined as the probability that the null hypothesis $\underline{\delta }=0$ will be rejected for at least one‐dose group. In Table 1 we compare the operating characteristics of this single‐stage Dunnett design with corresponding operating characteristics of stage‐wise MAMS designs that utilize three different multiplicity‐adjusted *P*‐values (Bonferroni, Simes, or Dunnett), and with the cumulative MAMS design, under a range of treatment differences from placebo for the three dose groups. These adaptive designs are conducted over two equally spaced stages in Table 1A and over three equally spaced stages in Table 1B. The adaptation occurs at the end of stage 1 and consists of early stopping if any dose group crosses an efficacy boundary, or dropping any dose group having an observed treatment effect that is worse than placebo. When doses are dropped their remaining sample sizes are reallocated in equal proportion to the remaining doses or placebo. The Bonferroni, Simes, and Dunnett stage‐wise MAMS procedures combine multiplicity‐adjusted *P*‐values derived from the Student's t distribution in accordance with Equation (A8) of Appendix A2. All table entries are based on 10 000 simulated trials. The value of α_*j*_ spent at each stage *j* to obtain the efficacy stopping boundaries is derived from the Lan and DeMets, O'Brien‐Fleming type, error spending function.^15^ For the stage‐wise MAMS designs these are the usual two‐arm group sequential boundaries, obtained as solutions to Equations (A11) and (A12) of Appendix A3. For the cumulative MAMS design, these are multiplicity adjusted multiarm group sequential boundaries, derived as shown in equations (A5) and (A6) of Appendix A2. However, as recommended by Wason et al,^17^ these multiarm boundaries, *b*_*j*_, are further transformed by the formula

(8) $$b_{ij}^{*}=\sqrt{{\hat{ℐ}}_{ij}}T_{d_{ij}}^{-1}\left(\Phi \left(\frac{b_{j}}{\sqrt{{\hat{ℐ}}_{ij}}}\right)\right),$$

to adjust for possible biases in small samples due to estimating the unknown $\sigma_{i}^{2}$ for each treatment *i* in the compuation of the test statistic. Here

(9) $${\hat{ℐ}}_{ij}=n_{0j}{\left({\hat{\sigma }}_{0j}^{2}+\frac{{\hat{\sigma }}_{ij}^{2}}{\lambda_{i}}\right)}^{-1},$$

is the estimated Fisher information about δ_*i*_ at stage *j*, ${\hat{\sigma }}_{i}^{2}$ is the estimated variance of the response to treatment *i*, based on cumulative data up to and including stage *j*, and $T_{d_{ij}}^{-1}$ is the inverse of the Student's t distribution with degrees of freedom *d*_*ij*_=*n*_0*j*_+*n*_*ij*_−1. This adjustment to the boundaries allows us to use estimated Fisher information in place of the unknown actual Fisher information without inflating the type‐1 error. The last rows of Table 1 show that this adjustment preserves the FWER, albeit slightly conservatively. We have verified that if the simulations are performed with the actual Fisher information, the FWER is exactly 0.025, thereby demonstrating that, in the absence of any large sample approximations, the adaptive cumulative MAMS design exhausts the entire α.

For the scenarios considered here, the adaptive cumulative MAMS design dominates the other designs with respect to power. Furthermore among the three stage‐wise MAMS methods displayed in Table 1, the methods that utilize the Bonferroni or Simes adjustments have considerably lower power than the method that utilizes the Dunnett adjustment. The power gains of the cumulative MAMS design over the other designs are more pronounced for heterogeneous treatment effects compared to homogeneous treatment effects. For example, it is seen from Table 1A for two‐stage designs where $\underline{\delta }=(0,0,0.187)$, that the cumulative MAMS design produces 6% more power than the stage‐wise MAMS design using Dunnett *P*‐values, 13% more power than the stage‐wise MAMS design using Simes *P*‐values, 14% more power than the stage‐wise MAMS design using Bonferroni *P*‐values, and 7% more power than the single‐stage Dunnett design.

It is interesting to observe that even in the homogeneous case where $\underline{\delta }=(0.187,0.187,0.187)$ the stage‐wise MAMS design using Dunnett *P*‐values has 2% less power than the cumulative MAMS design. This would appear to contradict the results of Section 4 where there is essentially no difference in power between stage‐wise and cumulative MAMS designs when the δ values are all equal. The explanation is that the designs in Section 4, unlike the SOCRATES‐REDUCED designs, do not include early stopping. The presence of early stopping boundaries causes a loss of power for stage‐wise MAMS relative to cumulative MAMS.

Table 1B displays similar results for three‐stage designs. Three‐stage designs, however, have the additional advantage of lower average sample sizes due to the possibility of early stopping. This is seen in Table 2

**Table 2:** Two‐stage vs three‐stage comparisons for cumulative multiarm multistage (MAMS)

	Power (std error)		Average Sample Size	
$\underline{\delta }$ (with σ=0.52)	Two‐Stage	Three Stage	Two‐Stage	Three‐Stage
(0.187, 0.187, 0.187)	0.805 (.004)	0.806 (0.004)	360	336
(0, 0.187, 0.187)	0.768 (.004)	0.773 (.004)	366	343
(0, 0, 0.187)	0.657 (.005)	0.647 (.005)	370	343
(0, 0, 0)	0.023 (.001)	0.023 (.001)	339	323

We noted at the end of Section 4.2 that the stricter the criterion for dropping doses at the end of stage 1, the greater the gain in power for cumulative MAMS over stage‐wise MAMS designs. It would be interesting to determine whether this result holds also for the SOCRATES‐REDUCED designs. In Table 3 we explore this conjecture for two‐stage designs with three different configurations for $\underline{\delta }$. In Table 3A, $\underline{\delta }=(0.187,0.187,0.187)$. In Table 3B, $\underline{\delta }=(0,0.187,0.187)$. In Table 3C, $\underline{\delta }=(0,0,0.187)$. In each table we use three progressively stricter criteria for dropping treatments—$\text{any}\;{\hat{\delta }}_{i1}<0$ in row 1, $\text{any}\;{\hat{\delta }}_{i1}<-\sigma$ in row 2, and $\text{any}\;{\hat{\delta }}_{i1}<-2\sigma$ in row 3.

**Table 3:** Power gains for adaptive cumulative multiarm multistage (MAMS) over adaptive stage‐wise MAMS

(A) *P*(*CUMUL*)−*P*(*STAGE*):(δ_1_,δ_2_,δ_3_)=(0.187,0.187,0.187) and σ=0.52			
Dose Dropping	Multiplicity‐adjusted *P*‐values for stage‐wise MAMS		
Criterion	Bonferroni	Simes	Dunnett
Any ${\hat{\delta }}_{i1}<0$	7.7%	1.8%	2.1%
Any ${\hat{\delta }}_{i1}<-\sigma$	8.5%	1.9%	2.2%
Any ${\hat{\delta }}_{i1}<-2\sigma$	7.3%	2.1%	1.5%
**(B) *P*(*CUMUL*)−*P*(*STAGE*):(δ_1_,δ_2_,δ_3_)=(0,0.187,0.187) and σ=0.52**			
**Dose Dropping**	**Multiplicity‐adjusted *P*‐values for stage‐wise MAMS**		
**Criterion**	**Bonferroni**	**Simes**	**Dunnett**
Any ${\hat{\delta }}_{i1}<0$	10.1%	5.5%	3.9%
Any ${\hat{\delta }}_{i1}<-\sigma$	15.7%	12.7%	9.2%
Any ${\hat{\delta }}_{i1}<-2\sigma$	15.3%	10.7%	7.9%
**(C) *P*(*CUMUL*)−*P*(*STAGE*):(δ_1_,δ_2_,δ_3_)=(0,0,0.187) and σ=0.52**		
**Dose Dropping**	**Multiplicity‐adjusted *P*‐values for stage‐wise MAMS**		
**Criterion**	**Bonferroni**	**Simes**	**Dunnett**
Any ${\hat{\delta }}_{i1}<0$	13.6%	13.1%	6.1%
Any ${\hat{\delta }}_{i1}<-\sigma$	21.0%	20.3%	14.3%
Any ${\hat{\delta }}_{i1}<-2\sigma$	17.7%	16.5%	11.5%

In each table, for each design, a pattern emerges whereby *P*(*CUMUL*)−*P*(*STAGE*) increases in moving from row 1 to row 2 and then decreases in moving from row 2 to row 3. A similar pattern was observed for the three‐stage designs. We are unable to find an explanation for this behavior. It is note‐worthy however, that the gains in power increase substantially with increasing heterogeneity of the δ values. For example, in Table 3C the value of *P*(*CUMUL*)−*P*(*STAGE*) can be as high as 21% for Bonferroni, 20.3% for Simes and 14.3% for Dunnett.

## 6 DISCUSSION

The usual practice in clinical drug development has been to first run a phase 2 trial with multiple doses, and then run a separate two‐arm phase 3 trial in which the best dose from phase 2 is compared to a control arm. Adaptive designs combine phase 2 and phase 3 into a single integrated trial and thereby utilize fewer patient resources and shorten the time required to identify and market efficacious medical products. To be acceptable for regulatory submissions such designs must have strong control of FWER. Both the stage‐wise MAMS and the cumulative MAMS designs have this property.

In stage‐wise MAMS designs, FWER control is achieved by constructing the test statistic as a weighted combination of inverse normal multiplicity‐adjusted *P*‐values from the incremental data at each stage, and monitoring this statistic with respect to the classical two‐arm group sequential boundaries. Since the weights are prespecified, this test statistic has the cannonical distribution of the usual two‐sample Wald or score statistic under the global null hypothesis, even if the sample size is reestimated in the course of the trial. Additionally, closed testing is implemented to identify the active treatment arms. In cumulative MAMS designs, strong FWER control is achieved by constructing a separate cumulative Wald or score statistic for each pairwise comparison and monitoring it with respect to group sequential boundaries that are adjusted for testing multiple treatment arms. Although these boundaries provide strong control of the FWER in the presence of arbitrary or unplanned treatment selection, they can be sharpened through step‐down closed testing and preservation of conditional error rates as described in Section 2 and Appendix A2. The sharpened boundaries provide additional flexibility to alter the sample size. Thus the stage‐wise and cumulative MAMS designs provide the same degree of flexibility to make adaptive changes to an ongoing design. There is, however, a fundamental difference in the handling of multiplicity by the two methods. In stage‐wise MAMS the multiplicity is incorporated into the adjusted *P*‐values whereas in cumulative MAMS it is incorporated into the group sequential boundaries.

We have compared the stage‐wise MAMS and cumulative MAMS approaches in a systematic manner under different configurations of the treatment effects and decision rules for dropping arms. Our first investigation, in Section 4.1, was for two treatment arms vs a common control arm with no treatment selection and no early stopping. In this simple setting it was possible to compare the two designs analytically and thus determine with great accuracy that only in the homogeneous case where δ_1_=δ_2_ does the stage‐wise MAMS design have greater power than the cumulative MAMS design. Moreover the power differential for this configuration of δ is at most 0.2%. For all other configurations the cumulative MAMS design has greater power with the power differential increasing as the δ values separate, and reaching 5% when the δ values are farthest apart. Next, in Section 4.2, we investigated the case of three treatment arms vs a common control arm, with treatment selection at the end of stage one but no early stopping. This investigation was by simulation and demonstrated greater power gains, up to 11% for cumulative MAMS designs over stage‐wise MAMS designs. As before, the power gains increased with greater heterogeneity among the δ values. Finally, in Section 5 we simulated two and three‐stage designs with dose selection as well as sample size reestimation for the SOCRATES‐REDUCED clinical trial. Here too the cumulative MAMS designs had greater power than the stage‐wise MAMS designs, with power gains that increased substantially with greater heterogeneity among the δ values. For example, for $\underline{\delta }=(0,0,0.187)$ one could obtain a 14.3% power gain for cumulative MAMS over stage‐wise MAMS with Dunnett‐adjusted *P*‐values, a 20.3% power gain over stage‐wise MAMS with Simes‐adjusted *P*‐values and a 21% power gain over stage‐wise MAMS with Bonferroni‐adjusted *P*‐values.

While the large power gains for cumulative MAMS designs over stage‐wise MAMS designs shown here have not been shown previously, they are consistent with results published in Koenig et al,^12^ Friede and Stallard^18^ and Magirr et al.^7^ Koenig et al^12^ and Friede and Stallard^18^ showed a benefit for the adaptive Dunnett test over the *P*‐value combination test for two‐stage designs with treatment selection but no early stopping or sample size reestimation. Magirr et al^7^ investigated two and three‐stage designs with treatment selection, early stopping and sample size reestimation, and showed a benefit for the “CE‐SB” and “CE‐AP” designs that utilize cumulative statistics and recompute multiplicity adjusted stopping boundaries through use of conditional error rates to control the FWER, over the “PC‐SB” designs that control the FWER through inverse normal combination of adjusted *P*‐values.

Even small gains in power can translate into huge sample size savings for cumulative MAMS designs over stage‐wise MAMS designs. For example, it is seen from Table 1B that, for a sample size of 388, if $\underline{\delta }=(0,0,0.187)$ the cumulative MAMS design has 64.7% power while the stage‐wise MAMS design has 59.2% power. In order for the stage‐wise MAMS design to also have 64.7% power, 448 subjects would be needed. Furthermore, as can be seen from Table 2, the average sample size of the cumulative MAMS design in this three‐stage early‐stopping setting is 343 subjects. We have determined in a separate simulation that the corresponding average sample size of the stage‐wise MAMS design is 424 subjects.

It was conjectured by a reviewer that the power advantage of the cumulative MAMS design over the stage‐wise MAMS design in Section 5 might be due to the specific sample‐size increase rule utilized in our simulations. This rule, which might be termed “proportional upscaling,” requires that the initially specified total sample size not be reduced when arms are dropped at an interim analysis. Instead the sample size that would have been assigned to the dropped arms is reallocated to continuing arms, in proportion to the original allocation ratios. To check the validity of this conjecture we resimulated the designs in Table 1A without proportional upscaling. In Table 4 we display power and sample size comparisons for the two‐stage SOCRATES design in which the unallocated sample sizes of the dropped arm are not reassigned to the arms that continue. As can be seen, these results are qualitatively similar to those of Table 1A. Thus the power advantage of the cumulative MAMS design appears to hold with or without proportional upscaling.

**Table 4:** Power comparisons without proportional upscaling (10 000 simulated trials)

	Power (SE)				
	Single‐	Adaptive Stage‐Wise Multiarm Multistage			Adaptive
	Stage				Cumulative
$\underline{\delta }$ (with σ=0.52)	Dunnett	Bonferroni	Simes	Dunnett	MAMS
(0.187, 0.187, 0.187)	0.804 (.004)	0.714 (.005)	0.775 (.004)	0.771 (.004)	0.789 (.004)
(0, 0.187, 0.187)	0.731 (.004)	0.584 (.005)	0.629 (.005)	0.656 (.005)	0.692 (.005)
(0, 0, 0.187)	0.591 (.005)	0.380 (.005)	0.398 (.005)	0.453 (.005)	0.502 (0.005)
(0, 0, 0)	0.025 (.002)	0.012 (.001)	0.015 (.001)	0.017 (.001)	0.024 (.002)
Drop any treatment *i* at stage 1 if corresponding ${\hat{\delta }}_{i1}<0$					

The conclusions we draw from the results presented in this paper are as follows:

1. Cumulative MAMS designs appear to be more powerful than stage‐wise MAMS design except in the homogeneous case where all the δ values are the same.
2. For the special case of two active treatments, with no treatment selection or sample size increase, analytical comparisons were possible. They revealed that when δ_1_=δ_2_ there is a small advantage for the stage‐wise MAMS design over the cumulative MAMS design, but it disappears as the two δs begin to diverge. It is thus entirely plausible that the same effect is present in the more complex setting of multiple doses, multiple looks and sample size reestimation considered in Sections 4.2 and 5. If present, however, the effect is too small to be detected in an experiment involving 10 000 simulated trials.
3. The magnitude of the power gain of cumulative MAMS designs over stage‐wise MAMS designs can be substantial and increases with increasing heterogeneity of the δ values.
4. Our results are based on a reasonably exhaustive exploration of the parameter space for three active treatment arms under specific decision rules for treatment selection, sample size reestimation and early stopping. We cannot claim that they hold for all possible adaptive designs. Nevertheless the designs that we have considered here are ones that are likely to adopted in practice. For other designs it is recommended to explore the operating characteristics of the two approaches by simulation using the tools we have discussed here.

We tried to ascertain why the cumulative MAMS approach was more powerful than the stage‐wise MAMS approach. We have three conjectures.

1. For the special case of two active doses with no early stopping or dropping of doses we were able to obtain explict power functions for the two methods in Section 4.1 and thereby demonstrate that the cumulative MAMS test, unlike the stage‐wise MAMS test is based on sufficient statistics
2. When there is no sample size reestimation the multiplicity‐adjusted cumulative MAMS boundaries are consonant. That is, although these boundaries have been constructed under the global null hypothesis *H*_0_, any elementary hypothesis $H_{0}^{i}$ for which *w*_*ij*_≥*b*_*j*_ can be rejected without loss of FWER control. In contrast, in order to reject $H_{0}^{i}$ in the stage‐wise MAMS approach, one must always go through the entire closed testing procedure
3. If treatments are dropped at an interim look in the cumulative MAMS design it is possible gain efficiency through boundary recomputation in conjunction with closed testing. Specifically, in the two‐stage cumulative MAMS design, the final critical value for testing $H_{0}^{I}$ is adjusted from *b*_*I*2_ to $b_{I2}^{*}$ by imposing the Müller and Schäfer condition^11^ through Equation (2). Although not shown here, we have verified that $b_{I2}^{*}\leq b_{I2}$ so that this adjustment confers an advantage on the group sequential approach that is not available to the *P*‐value combination approach.

We have not been able to explain why *P*(*CUMUL*)−*P*(*STAGE*) increases with increasing heterogeneity of the δ values. We are also unable to explain why *P*(*CUMUL*)−*P*(*STAGE*) first increases with increasing conservatism of the rule for dropping arms and then decreases. This phenomenon is manifest in every column of Table 3. We believe that this behavior is worth further investigation.

Throughout this paper we have utilized score statistics for monitoring the data and performing the hypothesis tests. We assumed in Section 2 that the scores are normally distributed with independent increments. These distributional properties hold exactly for normal data with known variance and asymptotically for all other settings in which the variance is estimated by maximum likelihood methods.^14^ We showed in Section 5, Equations (8) and (9), how one might use the t‐distribution to transform the cumulative MAMS boundaries and thereby obtain type‐1 error control for the case of normal data with unknown variance. We did not examine the accuracy of the asymptotic distributions when the underlying data are binomial or have time‐to‐event end points. In this regard the stage‐wise MAMS approach, though not as powerful as the cumulative MAMS approach, might be more robust since one can combine *P*‐values that are adjusted for multiplicity by nonparametric methods like the Bonferroni and Simes method rather than resort to normal approximations. On the other hand if convergence of the score statistics to asymptotic normality with independent increments was in doubt one could set the nominal type‐1 error of the cumulative MAMS design to be smaller than the desired α, say α/2, so as to ensure that the actual type‐1 error would be controlled at level‐α. The huge power advantage that the cumulative MAMS design enjoys over stage‐wise MAMS designs that utilize multiplicity adjusted nonparametric *P*‐values, as evidenced by Table 3 of Section 5, would probably not be offset even by extreme conservatism in the choice of the nominal α. This reasoning would not, however, be applicable if we were interested in testing multiple endpoints rather than testing multiple treatment arms. The multiarm problem is amenable to cumulative MAMS designs because the interarm correlation structure can be determined exactly from the treatment to control allocation ratio. The correlations between multiple endpoint must be estimated from the data and hence are subject to sampling error. Thus for multiple endpoint problems the stage‐wise MAMS methods that utilize the nonparametric Simes or Bonferroni adjustments to control the multiplicity might have an advantage over the cumulative MAMS methods that rely on large‐sample approximations. This is a topic for further investigation.

Another topic for further investigation is parameter estimation at the end of the trial. Bias reduction methods were investigated by Posch et al^5^ for stage‐wise MAMS designs with dose selection but no sample size adaptation. For two‐arm group sequential designs with adaptive sample size reestimation, methods have been developed by Gao et al,^19^ Brannath et al,^20^ and Mehta et al.^21^ There has been some recent work on unbiased point estimates in phase 2‐3 trials by Bowden and Glimm,^22^ Robertson et al,^23^ and Stallard and Kimani.^24^ Magirr et al^25^ have proposed simultaneous confidence intervals that are compatible with closed testing in adaptive designs. Further study is needed to understand how these methods may be incorporated into the general framework presented here.

## Appendix A1 ANALYTICAL COMPARISON WITH TWO ACTIVE DOSES AND TWO STAGES

Patients are randomized equally between the three arms of the study and each patient's response is normally distributed with σ^2^=1. The control arm has a mean of zero and treatment *i* has mean δ_*i*_, *i*=1,2. The null hypothesis corresponding to the treatment *i* is $H_{0}^{i}:\delta_{i}=0$. In this section we will test the global null hypothesis $H_{0}=H_{0}^{1}\cap H_{0}^{2}$ against the one‐sided alternative that δ_*i*_>0 for at least one *i*=1,2 . There will be no early stopping for efficacy, no dropping of treatments and no adaptive sample size reestimation.

### 1 (a) Analytical Power for Cumulative MAMS

Denote by *P*(*CUMUL*) the probability of rejecting *H*_0_ when the true treatment effect is $\underline{\delta }=(\delta_{1},\delta_{2})$. Since there is no early stopping, the first stage boundary *b*_1_ is ∞. Let *b*_2_ denote the second stage boundary. Let *f*_1_(*w*_11_,*w*_21_) be the probability density function of ${\underline{W}}_{1}=(W_{11},W_{21})$, the stage 1 score statistics. Let *f*_(2)_(*w*_1(2)_,*w*_2(2)_) be the probability density function of ${\underline{W}}_{\left(2\right)}=(W_{1(2)},W_{2(2)})$, the incremental stage 2 score statistics. These densities are multivariate normal with means, variances and covariance structures that depend on $\underline{\delta }$ as specified in Section 2. For notational convenience, however, we do not express, explicitly, the dependence of these density functions on $\underline{\delta }$.

(A1) $$\begin{array}{ll} P(CUMUL) & =P_{\underline{\delta }}\left(\max\{{\underline{W}}_{2}\}\geq b_{2}\right)\\ & =\overset{\infty }{\underset{w_{11}=-\infty }{\int }}\overset{\infty }{\underset{w_{21}=-\infty }{\int }}P_{\underline{\delta }}(\max\{{\underline{W}}_{2}\}\geq b_{2}|w_{11},w_{21})f_{1}(w_{11},w_{21})dw_{21}dw_{11}\\ & =1-\overset{\infty }{\underset{w_{11}=-\infty }{\int }}\overset{\infty }{\underset{w_{21}=-\infty }{\int }}P_{\underline{\delta }}(\max\{{\underline{W}}_{2}\}<b_{2}|w_{11},w_{21})f_{1}(w_{11},w_{21})dw_{21}dw_{11}\\ & =1-\overset{\infty }{\underset{w_{11}=-\infty }{\int }}\overset{\infty }{\underset{w_{21}=-\infty }{\int }}P_{\underline{\delta }}\left(W_{1(2)}<b_{2}-w_{11}\cap W_{2(2)}<b_{2}-w_{21})\right)f_{1}(w_{11},w_{21})dw_{21}dw_{11}\;\\ & =1-\overset{\infty }{\underset{-\infty }{\int }}\overset{\infty }{\underset{-\infty }{\int }}\left(\overset{b_{2}-w_{11}}{\underset{w_{1(2)}=-\infty }{\int }}\overset{b_{2}-w_{21}}{\underset{w_{2(2)}=-\infty }{\int }}f_{\left(2\right)}\left(w_{1(2)},w_{2(2)}\right)dw_{2(2)}dw_{1(2)}\right)f_{1}(w_{11},w_{21})dw_{21}dw_{11} \end{array}$$

The fourth line of the above equation utilizes the fact that ${\underline{W}}_{j}$ has independent increments.

### 2 (b) Analytical Power for Stage‐Wise MAMS

We first evaluate the incremental *P*‐values for the two stages. The stage 1 *P*‐value is evaluated as

$$P_{1}=P_{\underline{0}}(\max\{{\underline{W}}_{1}\}\geq \max\{{\underline{w}}_{1}\})=1-P_{\underline{0}}(W_{11}<\max\{{\underline{w}}_{1}\}\cap W_{21}<\max\{{\underline{w}}_{1}\})\;.$$

The stage 2 *P*‐value, *P*_(2)_, is computed from the incremental data obtained after the interim analysis. Letting $\max\{{\underline{W}}_{\left(2\right)}\}=\max\{W_{1(2)},W_{2(2)}\}$, we have

$$P_{\left(2\right)}=P_{\underline{0}}(\max\{{\underline{W}}_{\left(2\right)}\}\geq \max\{{\underline{w}}_{\left(2\right)}\})=1-P_{\underline{0}}(W_{1(2)}<\max\{{\underline{w}}_{\left(2\right)}\}\cap W_{2(2)}<\max\{{\underline{w}}_{\left(2\right)}\})\;.$$

Since there is no early stopping, *H*_0_ is rejected if

$$h_{1}z_{p_{1}}+h_{2}z_{p_{\left(2\right)}}\geq z_{\alpha }\;,$$

where *z*_γ_=Φ^−1^(1−γ). The power of the *P*‐value combination test to reject *H*_0_ is

(A2) $$\begin{array}{ll} P(STAGE) & =P_{\underline{\delta }}\left(h_{1}z_{p_{1}}+h_{2}z_{p_{\left(2\right)}}\geq z_{\alpha }\right)\\ & =\overset{\infty }{\underset{w_{11}=-\infty }{\int }}\overset{\infty }{\underset{w_{21}=-\infty }{\int }}P_{\underline{\delta }}\left(z_{p_{\left(2\right)}}\geq \frac{z_{\alpha }-h_{1}z_{p_{1}}}{h_{2}}\left|\right.w_{11},w_{21}\right)f_{1}(w_{11},w_{21})dw_{21}dw_{11}\\ & =\overset{\infty }{\underset{w_{11}=-\infty }{\int }}\overset{\infty }{\underset{w_{21}=-\infty }{\int }}P_{\underline{\delta }}\left(p_{\left(2\right)}\leq 1-\Phi \left(\frac{z_{\alpha }-h_{1}z_{p_{1}}}{h_{2}}\right)\right)f_{1}(w_{11},w_{21})dw_{21}dw_{11} \end{array}$$

*P*(*STAGE*) can be further simplified for better comparison with *P*(*CUMUL*). Define the univariate function

$$F_{\left(2\right)}(x)=P_{\underline{0}}(W_{1(2)}\leq x\cap W_{2(2)}\leq x)\;.$$

Then

(A3) $$\begin{array}{ll} p_{\left(2\right)}\leq 1-\Phi (\frac{z_{\alpha }-h_{1}z_{p_{1}}}{h_{2}}) & \Leftrightarrow P_{\underline{0}}(\max\{{\underline{W}}_{\left(2\right)}\}\geq \max\{{\underline{w}}_{2}\})\leq 1-\Phi (\frac{z_{\alpha }-h_{1}z_{p_{1}}}{h_{2}})\\ & \Leftrightarrow 1-P_{0}(W_{1(2)}\leq \max\{{\underline{w}}_{\left(2\right)}\}\cap W_{2(2)}\leq \max\{{\underline{w}}_{\left(2\right)}\})\leq 1-\Phi (\frac{z_{\alpha }-h_{1}z_{p_{1}}}{h_{2}})\\ & \Leftrightarrow F_{\left(2\right)}(\max\{{\underline{w}}_{\left(2\right)}\})\geq \Phi (\frac{z_{\alpha }-h_{1}z_{p_{1}}}{h_{2}}) \end{array}$$

Then, substituting Equation (A3) into Equation (A2) we have

(A4) $$\begin{array}{ll} P(STAGE) & =\overset{\infty }{\underset{w_{11}=-\infty }{\int }}\overset{\infty }{\underset{w_{21}=-\infty }{\int }}P_{\underline{\delta }}\left(F_{\left(2\right)}(\max\{{\underline{w}}_{\left(2\right)}\})\geq \Phi \left(\frac{z_{\alpha }-h_{1}z_{p_{1}}}{h_{2}}\right)\right)f_{1}(w_{11},w_{21})dw_{21}dw_{11}\\ & =\overset{\infty }{\underset{w_{11}=-\infty }{\int }}\overset{\infty }{\underset{w_{21}=-\infty }{\int }}P_{\underline{\delta }}\left(\max\{{\underline{w}}_{\left(2\right)}\}\geq F_{\left(2\right)}^{-1}\left(g\right)\right)f_{1}(w_{11},w_{21})dw_{21}dw_{11}\\ & =1-\overset{\infty }{\underset{-\infty }{\int }}\overset{\infty }{\underset{-\infty }{\int }}\left(\overset{F_{\left(2\right)}^{-1}(g)}{\underset{w_{1(2)}=-\infty }{\int }}\overset{F_{\left(2\right)}^{-1}(g)}{\underset{w_{2(2)}=-\infty }{\int }}f_{\left(2\right)}\left(w_{1(2)},w_{2(2)}\right)dw_{2(2)}dw_{1(2)}\right)f_{1}(w_{11},w_{21})dw_{21}dw_{11}, \end{array}$$

where $g=\Phi \left(\frac{Z_{\alpha }-h_{1}Z_{p_{1}}}{h_{2}}\right)$ is a function of the maximum of (*w*_11_,*w*_21_) through *p*_1_.

## Appendix A2 EXTENDING CUMULATIVE MAMS TO *J* > 2 STAGES

Consider a *J*‐stage multiarm group sequential design in which α_*j*_ is spent at stage *j* in accordance with some α‐spending function such that $\sum_{j=1}^{J}\alpha_{j}=\alpha$. Then the corresponding efficacy boundaries (*b*_1_,*b*_2_,…*b*_*j*_) satisfy the requirements

(A5) $$P_{0}(\max\{{\underline{W}}_{1}\}\geq \max\{w_{1}\})=\alpha_{1}$$

and for *j*=2,3,…*J*,

(A6) $$\alpha_{j-1}+P_{0}\left(\overset{j-1}{\underset{l=1}{⋂}}(\max\{{\underline{W}}_{l}\}<b_{l})\cap \max\{{\underline{W}}_{j}\}\geq b_{j}\right)=\alpha_{j}\;.$$

We have shown in Reference 9 how to compute such boundaries. Now suppose we perform a one‐time dose selection and sample size reestimation at some stage *q*<*J*. Let 𝒟={1,2,…D} denote the indices of the *D* treatments and S⊆𝒟 denote the indices of the treatments selected for further testing at stages *q*+1,*q*+2,…*J*. We wish to test $H_{0}^{i}$ for all *i*∈*S* while maintaining strong control of FWER at level‐α. Therefore, based on the closed testing principle, each $H_{0}^{i}$ may only be rejected if, for all I⊆𝒟 such that *i*∈*I*, $H_{0}^{I}=\cap_{g\in I}H_{0}^{g}$ is rejected by a valid local level‐α test. The following two‐step procedure may be used to construct the local level‐α test of $H_{0}^{I}$.

Step 1 Compute *J* new group sequential boundaries (*b*_*I*1_,*b*_*I*2_,…*b*_*IJ*_) that are suitable for making ||*I*||≤*D* treatment comparisons to the common control arm. These boundaries must satisfy
  $$P_{0}(\max\{{\underline{W}}_{I1}\}\geq b_{I1})=\alpha_{1}$$
  and for *j*=2,3,…*J*,
  $$\alpha_{j-1}+P_{0}\left(\overset{j-1}{\underset{l=1}{⋂}}(\max\{{\underline{W}}_{Il}\}<b_{Il})\cap \max\{{\underline{W}}_{Ij}\}\geq b_{Ij}\right)=\alpha_{j}\;,$$
  where ${\underline{W}}_{Ij}=\{W_{ij};i\in I\}$. If $\max\{{\underline{w}}_{Iq}\}\geq b_{Iq}$, $H_{0}^{i}$ is rejected. Otherwise we proceed to Step 2.
Step 2 After examining the stage q data a subset S⊆𝒟 consisting of ||*S*|| treatments is selected for testing at stages *q*+1,*q*+2,…*J*, possibly accompanied by an increase in the sample sizes of the selected doses. Let *I*_*S*_=*I*∩*S*. In order to obtain a valid level‐α test of *H* ^*I*^ while accommodating this adaptation, we must replace the future boundaries (*b*_*I*,*q*+1_,*b*_*I*,*q*+2_…*b*_*IJ*_) with updated boundaries ($b_{I,q+1}^{*},b_{I,q+2}^{*}\ldots b_{IJ}^{*}$) that satisfy the Müller and Schäfer criterion^11^ for preserving the conditional type‐1 error. Thus these updated boundaries must satisfy the relationship
  (A7) $$P_{0}\left(\overset{\left||S|\right|}{\underset{l=q+1}{⋃}}\max\{{\underline{W}}_{I_{S}l}^{*}\}\geq b_{Il}^{*}|{\underline{w}}_{Iq}\right)=P_{0}\left(\overset{J}{\underset{l=q+1}{⋃}}\max\{{\underline{W}}_{Il}\}\geq b_{Il}|{\underline{w}}_{Iq}\right),$$
  where ${\underline{W}}_{I_{S}l}^{*}=\{W_{ql}^{*}:q\in I_{S}\}$ and the ‘∗′ indicates that the sample size of the stage *l* statistic $W_{I_{S}l}^{*}$ has been altered. We reject $H_{0}^{I}$ if, for any *l*∈{*q*+1,*q*+2,||*S*||}, $\max\{{\underline{w}}_{I_{S}l}^{*}\}\geq b_{Il}^{*}$. The method of Ghosh et al^9^ can be applied to Equation (A7) to obtain $\left(b_{I,q+1}^{*},b_{I,q+2}^{*}\ldots b_{IJ}^{*}\right)$, possibly with a spending function for the remaining α that is different from the one that was selected initially. The details have been omitted for brevity.

## Appendix A3 EXTENDING STAGE‐WISE MAMS TO *J* > 2 STAGES

Recall that we can reject any elementary hypothesis $H_{0}^{i}$ only if the intersection hypothesis $H_{0}^{I}$ is rejected by a valid local level‐α test for all subsets I⊆𝒟 that contain *i*. To test $H_{0}^{I}$ at any stage *j* we require the multiplicity‐adjusted *P*‐values *P*_*I*(1)_,*P*_*I*(2)_,…*P*_*I*(*j*)_ where each *P*_*I*(*l*)_ utilizes only the *incremental* data of subjects enrolled between stages *l*−1 and *l*. These *P*‐values are transformed by the inverse normal function and combined with prespecified weights to form the stage‐wise test statistic

(A8) $$Z_{Ij}=h_{1j}\Phi^{-1}(1-p_{I(1)})+h_{2j}\Phi^{-1}(1-p_{I(2)})+\ldots +h_{jj}\Phi^{-1}(1-p_{I(j)}),$$

where, for *l*=1,2,…*j*,

$$h_{lj}=\frac{\sqrt{N_{\left(l\right)}/N_{J}}}{\sqrt{(N_{\left(1\right)}+N_{\left(2\right)}+\ldots +N_{\left(j\right)})/N_{J}}}\;,$$

*N*_*J*_ is the preplanned total sample size of the trial, and *N*_(*l*)_ is preplanned incremental number of patients to be enrolled between stages *l*−1 and *l*. Any valid multiplicity‐adjusted *P*‐values may be utilized in Equation (A8) for the test of $H_{0}^{I}$. Popular candidates include the t‐test based *P*‐values adjusted for multiplicity by the nonparametric Bonferroni and Simes procedures as shown in Reference 5. However, in order to make a meaningful comparison between the cumulative MAMS and the stage‐wise MAMS approaches, we will utilize *P*‐values that are derived from the maximum score statistic. In that case the multiplicity adjusted *P*‐values in Equation (A8) are given by

(A9) $$p_{I(l)}=P_{\underline{0}}\left(\max\{{\underline{W}}_{I(l)}\}\geq \max\{{\underline{w}}_{I(l)}\}\right),$$

where, for *l*=1,2,…*j*, ${\underline{W}}_{I(l)}$ is the vector of *incremental* score statistics contained in the subset I⊆𝒟 (or in the subset *I*∩*S* if *S*⊆*I* treatments have been selected for further testing by stage *l*). Thereby Equation (A8) is a weighted sum of inverse normal *P*‐values derived from Dunnett's test.^16^ To evaluate *P*_*I*(*l*)_ exactly we standardize the observed score to be

$$t_{i(l)}=\frac{w_{i(l)}}{\sqrt{{\hat{ℐ}}_{i(l)}}},$$

for all *i*∈*I*, where ${\hat{ℐ}}_{i(l)}$ is the estimated Fisher information from the incremental data between stage (*l*−1) and stage *l*. Define ${\underline{T}}_{I(l)}=\{t_{i(l)};i\in I\}$.Then the multiplicity adjusted Dunnett *P*‐value can be computed exactly as

(A10) $$p_{I(l)}=P_{H_{0}^{I}}\left(\max\{{\underline{T}}_{I(l)}\}\geq \max\{{\underline{t}}_{I(l)}\}\right),$$

where ${\underline{T}}_{I(l)}$ has a multivariate‐*T* distribution with mean $\underline{0}$, $n_{0(l)}+\sum_{i\in I}n_{i(l)}-||I||-1$ degrees of freedom, and a known correlation matrix that depends on the allocation ratios of the treatment arms to the control arm.

Since the *P*‐values in Equation (A8) are computed from independent cohorts of patients and are combined with prespecified weights whose sum of squares is 1, the statistic *Z*_*Ij*_ is *N*(0,1) with independent increments under the null hypothesis $H_{0}^{I}$ for all *j* and all *I*. Thus one can readily obtain efficacy boundaries *c*_1_,*c*_2_,…*c*_*J*_ such that

(A11) $$P_{H_{0}^{I}}(Z_{I1}\geq c_{1})=\alpha_{1}$$

and for *j*=2,3,…*J*,

(A12) $$\alpha_{j-1}+P_{H_{0}^{I}}\left(\overset{j-1}{\underset{l=1}{⋂}}(Z_{Il}<c_{l})\cap Z_{Ij}\}\geq c_{j}\right)=\alpha_{j}$$

by the usual methods for two‐arm group‐sequential designs.^2^ The α_*j*_s are obtained by specifying any suitable error spending function. The null hypothesis $H_{0}^{I}$ is rejected at the first *j* such that *Z*_*Ij*_≥*c*_*j*_. We reject $H_{0}^{i}$ with strong control of FWER if $H_{0}^{I}$ is rejected for all possible I⊆𝒟 with *i*∈*I*.
